# Treatment of non-small cell lung cancer: advances following the introduction of PET-CT and IMRT/VMAT

**DOI:** 10.1007/s00066-025-02377-0

**Published:** 2025-03-06

**Authors:** Julian Muster, Niklas Josua Alt, Marcus Edelmann, Mahalia Zoe Anczykowski, Carla Marie Zwerenz, Markus Anton Schirmer, Tobias Raphael Overbeck, Friederike Braulke, Manuel Guhlich, Rami El Shafie, Stefan Rieken, Martin Leu, Leif Hendrik Dröge

**Affiliations:** 1 Department of Radiotherapy and Radiation Oncology, University Medical Center Göttingen, Robert-Koch-Str. 40, 37075 Göttingen, Germany; 2 Göttingen Comprehensive Cancer Center (G-CCC), University Medical Center Göttingen, Von-Bar-Str. 2/4, 37075 Göttingen, Germany; 3 Department of Hematology and Medical Oncology, University Medical Center Göttingen, Robert-Koch-Str. 40, 37075 Göttingen, Germany

**Keywords:** Non-small cell lung cancer, Radiotherapy, Radiochemotherapy, Positron emission tomography-computed tomography, 3D-conformal radiotherapy, Intensity-modulated radiotherapy, Volumetric modulated arc therapy

## Abstract

**Purpose:**

In definitive radiotherapy/radiochemotherapy (RT/RCT) for localized non-small cell lung cancer (NSCLC), the introduction of positron-emission tomography (PET)-CT-based staging/RT planning and dynamic RT techniques (intensity-modulated radiotherapy, IMRT/volumetric modulated arc therapy, VMAT) were important innovations.

**Methods:**

We performed a retrospective study and compared clinical outcomes (1) in patients with PET-CT-based staging (*n* = 170) vs. conventional staging (*n* = 103) and (2) in patients with dynamic RT techniques (IMRT/VMAT; *n* = 99) vs. three-dimensional conformal radiotherapy (3D-CRT; *n* = 64).

**Results:**

We found improved survival with PET-CT vs. conventional staging. PET-CT patients vs. conventionally staged patients had higher applied RT doses, higher RT completion rates, and a higher rate of patients who received RCT vs. RT only. Additionally, we found higher rates of leukopenia and lung infections in PET-CT patients. When comparing RT techniques (IMRT/VMAT vs. 3D-CRT), there were no differences in survival. IMRT/VMAT patients had higher RT doses and higher rates of intensified concomitant chemotherapy (cisplatin/vinorelbine vs. low-dose cisplatin). IMRT/VMAT was associated with a reduction in pneumonitis and dermatitis.

**Conclusion:**

In summary, refined RT/RCT strategies with PET-CT and IMRT/VMAT enable the intensification of multimodal treatment. Reduction of toxicities with IMRT/VMAT widens the therapeutic window. The coincidence of intensified treatment, improved outcomes, and higher toxicity rates in PET-CT-staged patients emphasizes the need for a detailed risk–benefit assessment during planning and application of treatment modalities.

**Supplementary Information:**

The online version of this article (10.1007/s00066-025-02377-0) contains supplementary material, which is available to authorized users.

## Introduction

In 2020, lung cancer was diagnosed in approximately 22,600 women and 34,100 men in Germany (including adenocarcinoma in 44%, squamous cell cancer in 21%, and small cell lung cancer in 15%) [1]. Lung cancer-related deaths were documented in 17,066 women and 27,751 men [1]. Overall survival rates for lung cancer (independent of stage and histology) were about 25% in women and 19% in men [1]. In a cohort study with 851 patients who received curatively intended treatment for stage III non-small cell lung cancer (NSCLC) from 2010 to 2018, Meldgaard et al. reported that treatment and/or diagnosis in the more-contemporary study period (2016–2018) was associated with improved overall survival (OS) [2]. Refinement of imaging modalities for staging and radiotherapy (RT) planning purposes (i.e., positron-emission tomography–computed tomography, PET-CT) and of dynamic RT techniques have contributed relevantly to favorable outcomes [3–6].

In the present study, the focus was on the clinical outcomes of patients who had been treated with vs. without these refined strategies from 2008 to 2019. Patients who received definitive RT or radiochemotherapy (RCT) for localized NSCLC were retrospectively analyzed. We compared baseline, clinical, and treatment characteristics and outcomes (1) in patients with PET-CT-based staging vs. conventional staging and (2) in patients with dynamic RT techniques (intensity-modulated radiotherapy, IMRT/volumetric modulated arc therapy, VMAT) vs. three-dimensional conformal radiotherapy (3D-CRT).

## Materials and methods

### Study design

The study was approved by the local Ethics Committee of the University Medical Center Göttingen (protocol code 6/4/21, date of approval, 04/27/2021). Medical records of the local clinic were screened for patients with RT or RCT for NSCLC from January 2008 to December 2019. Here, 749 patients were identified. Patient selection and screening has previously been reported [7].

**Fig. 1:**
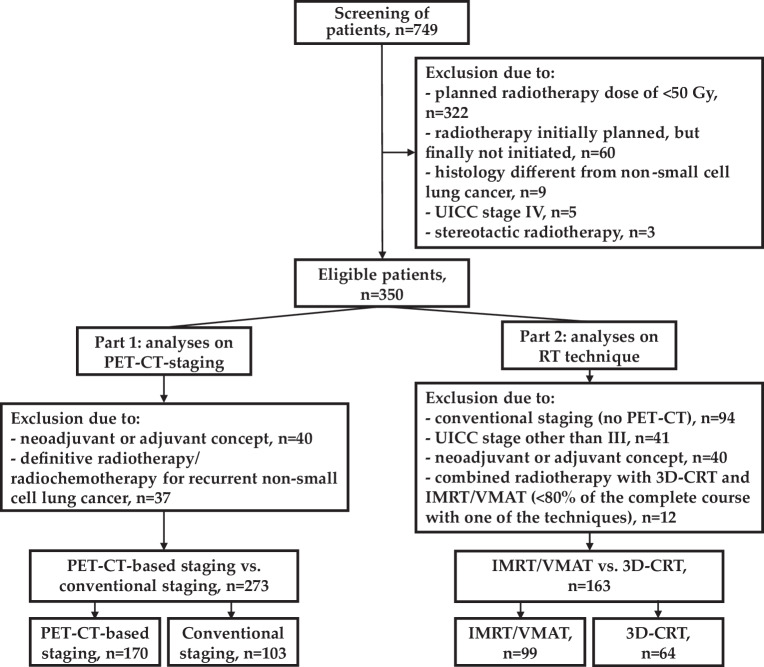
Flowchart. *PET-CT* positron-emission tomography–computed tomography, *RT* radiotherapy, *IMRT* intensity-modulated radiotherapy, *VMAT* volumetric modulated arc therapy, *3D-CRT* three-dimensional conformal radiotherapy

As the first subproject of this study (Fig. 1, left, part 1), we compared patients who received PET-CT-based staging vs. patients who received conventional staging. Here, we included only patients who received definitive RT or RCT in the case of a primary diagnosis of NSCLC. Based on previous studies in which distinct differences in definitive RT/RCT for primary diagnosis vs. treatment in case of recurrence (e.g., differences in locoregional vs. distant failure, prognostic factors [8]) became evident, we excluded patients with recurrent NSCLC. Finally, 273 patients were eligible for analyses on PET-CT-based staging (Fig. 1, left, part 1).

As the second subproject (Fig. 1, right, part 2), we compared patients who received IMRT/VMAT vs. 3D-CRT. We included patients who were staged with PET-CT as stage III NSCLC and received definitive RT or RCT. In patients who received combined RT with IMRT/VMAT and 3D-CRT, we excluded patients with no major technique identified for the RT course (neither IMRT/VMAT nor 3D-CRT; cut-off ≥ 80%). Patients who received ≥ 80% of the course either with IMRT/VMAT or with 3D-CRT were assigned to the respective study groups (Fig. 1, right, part 2).

### Staging and radiochemotherapy

Please see Alt et al. for a previous description of the RT/RCT procedures in the context of the local lung cancer center [7]. Procedures were based on current guidelines and recommendations [9–11]. Staging included an PET-CT scan (grade A recommendation for Union for International Cancer Control [UICC] stages IB–IIIB and optional in UICC stage IA according to current German S3 guidelines, recommendations 6.6 and 6.7 [9]) and brain MRI (recommendation 6.5 [9]) in patients with an existing treatment option. In cases with medical contraindications to PET-CT (e.g., diabetic metabolic status; S3 guidelines, recommendation 6.6 [9]) or in patients with a high tumor burden in whom a treatment delay was not acceptable (depending on the availability of PET-CT), conventional staging with bone scintigraphy and CT of the chest and abdomen was performed.

Please see Alt et al. for a previous description of RT methods [7]. Target volumes were delineated with consideration of RT guidelines [11, 12]. During the treatment period, involved-field RT was standardly applied. The margins for planning target volumes were set on an individual basis (study with comparison of PET-CT vs. conventional staging: *n* = 256 with 1 cm, *n* = 17 with 2 cm; study with comparison of IMRT/VMAT vs. 3D-CRT: *n* = 159 with 1 cm, *n* = 4 with 2 cm). The Eclipse treatment planning system was used (Varian Medical Systems, Palo Alto, USA). RT was performed with linear accelerators (Varian Medical Systems, Palo Alto, USA). Image guidance (3D-CRT at least weekly; IMRT/VMAT daily) included an electronic portal imaging device (EPID), on-board kilovolt imaging (OBI), and cone-beam CT (CBCT). 3D-CRT was applied with an individualized field arrangement with photon energies of 6 MeV and/or 20 MeV. If considered favorable, wedges were used. IMRT and VMAT (here, standard use of 6 MeV) were introduced into the clinic in 2009. In patients who were screened for this study, IMRT/VMAT were first used in 2011. The RT technique was chosen on an individual basis by the treating radiation oncologist. The organs at risk constraints were as follows: esophagus mean dose < 34 Gy [13]; lungs V20Gy ≤ 30–35% and mean dose ≤ 20–23 Gy [14]; spinal cord maximum dose 45 Gy (50 Gy for tumors/target volumes in close proximity to the spinal cord) [15]; brachial plexus maximum dose 60–66 Gy [16, 17]. Please see previous publication for details on local practice in systemic treatment [7].

### Endpoints and statistics

Please see Alt et al. [7]. Tumor stages were documented in accordance with TNM/UICC/American Joint Committee on Cancer (AJCC; study period 2008–2019; 6th, 7th, and 8th editions of TNM/UICC/AJCC [18–21]). For additional documentation (if required), the 8th edition was applied [7, 18–21]. Toxicity was documented in accordance with the Common Terminology Criteria for Adverse Events [22]. Endpoints included overall survival (OS; event: patient death from any cause), progression-free survival (PFS; events: patient death from any cause, recurrence [local and/or regional and/or distant]), locoregional progression-free survival (LRPFS; events: patient death from any cause, local and/or regional recurrence), and distant progression-free survival (DPFS; events: patient death from any cause, distant recurrence). Survival times were calculated from the day of histopathological diagnosis. Statistics (chi-square test, Kruskal–Wallis-test, and uni- and multivariable Cox regression analysis) were performed using Microsoft Excel (v2003, v2016; Microsoft Corporation, Redmond, Washington, USA), Statistica (v13.3; TIBCO Software Inc., Palo Alto, California, USA), and SPSS (v27-v29; IBM Corp., Armonk, N.Y., USA). Survival curves (with Kaplan–Meier statistics) were drawn using R (v4.1.0; R Foundation, Vienna, Austria) and the plugin KMWin (v1.53) [23]. *P*-values of < 0.05 were considered statistically significant.

## Results

### Staging with PET-CT vs. conventional staging

#### Baseline, clinical, and treatment characteristics

Parameters were compared in patients (*n* = 273) with PET-CT-based staging (*n* = 170) vs. patients with conventional staging (*n* = 103; Table 1). Patients staged with PET-CT presented with lower T stages (T1–2 vs. T3–4; *p* = 0.049). In patients with PET-CT, T1–2 was documented in 57/170 patients (33.5%) vs. T1–2 in 23/103 patients (22.3%) with conventional staging. Patients underwent PET-CT vs. conventional staging more frequently in the later (12/2013–12/2019; 70.6% vs. 16.5% of patients) than in the earlier (01/2008–11/2013; 29.4% vs. 83.5% of patients) treatment period (*p* < 0.01). IMRT/VMAT was more frequently applied (*p* < 0.01) in the PET-CT group (55.9% of patients) vs. the conventional staging group (15.5%). In patients with PET-CT vs. conventional staging, we found higher applied RT doses (median 66 Gy vs. 60 Gy; *p* < 0.01), a higher proportion of patients who completed radiotherapy (87.1% vs. 77.7%; *p* = 0.04), and higher proportion of patients who received ≥ 80% of the planned RT dose (93.5% vs. 82.5%; *p* < 0.01). There were no significant differences in target volume sizes or lung doses.

**Table 1:** Comparison of baseline, clinical, and treatment characteristics in patients with PET-CT-based staging vs. patients with conventional staging. Median (minimum–maximum) values or numbers of patients (percentage) are presented, if not otherwise specified

Parameter	Patients staged with PET-CT, *n* = 170	Patients with conventional staging, *n* = 103	*p*-value
Age	68.4 (32.5–89.2)	67.7 (40.4–87.8)	0.59^e^
Female	37 (21.8)	29 (28.2)	0.23^f^
Male	133 (78.8)	74 (72.8)	–
Karnofsky index (median, min–max)	90 (50–90)	90 (20–90)	0.38^f^
Charlson comorbidity index	4 (0–10)	4 (0–11)	0.89^f^
Stage cT1–2	57 (33.5)	23 (22.3)	0.049^f^
Stage cT3–4	113 (66.5)	80 (77.7)	–
Stage cN0–1	35 (20.6)	24 (23.3)	0.6^f^
Stage cN2–3	135 (79.4)	79 (76.7)	–
Squamous cell carcinoma	102 (60.0)	65 (63.1)	0.61^e^
Adenocarcinoma	63 (37.1)	33 (32.0)	–
Other types^a^	5 (2.9)	5 (4.9)	
Earlier treatment period, 01/2008–11/2013^b^	50 (29.4)	86 (83.5)	< 0.01^f^
Later treatment period, 12/2013–12/2019^c^	120 (70.6)	17 (16.5)	–
Radiotherapy technique: ≥ 80% of the course with intensity modulated radiotherapy (IMRT)/volumetric modulated arc therapy (VMAT)	95 (55.9)	16 (15.5)	< 0.01^e^
Radiotherapy technique: ≥ 80% of the course with 3D conformal radiotherapy (3D-CRT)	67 (39.4)	84 (81.6)	–
Radiotherapy technique: no major technique (neither IMRT/VMAT nor 3D-CRT; cut-off ≥ 80%)	8 (4.7)	3 (2.9)	
Radiotherapy only	26 (15.3)	24 (23.3)	0.1^f^
Radiochemotherapy	144 (84.7)	79 (76.7)	–
Concomitant cisplatin/vinorelbine	60 (41.7)	20 (25.3)	0.08^e^
Concomitant low-dose cisplatin	69 (47.9)	56 (70.9)	–
Other type of chemotherapy^d^	15 (10.4)	3 (3.8)	
Consolidation durvalumab	4 (2.4)	0 (0.0)	0.12^f^
Radiotherapy applied dose [Gy]	66 (12–70)	60 (2–66)	< 0.01^e^
Radiotherapy completed	148 (87.1)	80 (77.7)	0.04^f^
Radiotherapy ≥ 80% of the planned dose applied	159 (93.5)	85 (82.5)	< 0.01^f^
Clinical target volume [ml]	238.6 (18–1236)	243.2 (32–942)	0.82^e^
Planning target volume [ml]	718.7 (80–2244)	765.5 (155–2127)	0.54^e^
Lungs mean dose [Gy]	18.1 (3–28)	18.5 (1–31)	0.95^e^
Lungs V40Gy [%]	16.9 (0–37)	17.7 (0–36)	0.55^e^
Lungs V30Gy [%]	23.3 (0–43)	23.9 (0–48)	0.48^e^
Lungs V20Gy [%]	31.5 (0–51)	32.8 (0–70)	0.65^e^
Lungs V10Gy [%]	49.3 (6–86)	51.1 (0–87)	0.91^e^
Lungs V5Gy [%]	63.8 (9–100)	62.6 (0–93)	0.46^e^

^a^Further histologic types: not otherwise specified, *n* = 5; large cell carcinoma, *n* = 1; large cell neuroendocrine tumor, *n* = 1; pleomorphic carcinoma, *n* = 1; atypical carcinoid, *n* = 1; adenoid papillary differentiation, *n* = 1

^b^Initiation of radiotherapy/radiochemotherapy from 01/2008–11/2013 (allocation: median of the whole study group)

^c^Initiation of radiotherapy/radiochemotherapy from 12/2013–12/2019 (allocation: median of the whole study group)

^d^Other chemotherapy: concomitant chemotherapy, *n* = 6 (cisplatin/etoposide, *n* = 3; carboplatin/taxol, *n* = 1; cisplatin/pemetrexed, *n* = 1; vinorelbine/etoposide, *n* = 1); sequential chemotherapy, *n* = 12 (cisplatin/docetaxel, *n* = 5; cisplatin/pemetrexed, *n* = 2; carboplatin/paclitaxel, *n* = 2; cisplatin/navelbine, *n* = 1; carboplatin/gemcitabine, *n* = 1; pemetrexed alone, *n* = 1)

^e^Kruskal–Wallis test

^f^Pearson’s chi-square test

#### Toxicities

Toxicities were compared between patients staged with PET-CT vs. conventional staging (Table 2). Rates of lung infections were higher (≥ grade 2, *p* = 0.02; ≥ grade 3, *p* = 0.01) in patients with PET-CT (≥ grade 2, 25.9% of patients; ≥grade 3, 18.8%) vs. conventional staging (≥ grade 2, 13.6% of patients; ≥ grade 3, 7.8%). Additionally, rates of leukopenia were higher in patients with PET-CT vs. conventional staging (≥ grade 1, 66.5% vs. 48.5% of patients). It is of valid interest to assess whether differences in toxicities were specific for PET-CT-based staging. We compared baseline parameters (only in the case of relevance or differences in PET-CT-based vs. conventional staging; Table 1) between patients with lung infections ≥ grade 3 vs. < grade 3 and between patients with leukopenia ≥ grade 1 vs. grade 0. There were no significant differences in parameters for patients with lung infections (Suppl. Table S1; additional analysis of a possible association of leukopenia and lung infections based on previous studies [24]). In patients with leukopenia (Suppl. Table S2), there were higher proportions of patients in the later treatment period (12/2013–12/2019 vs. 01/2008–11/2013; *p* < 0.01; allocation of time periods: median of the whole study group), patients with RCT (vs. RT only; *p* < 0.01), higher applied RT dose (*p* < 0.01), and more patients with ≥ 80% of the planned RT dose applied (*p* < 0.01).

**Table 2:** Comparison of toxicities in patients with PET-CT(positron emission tomography-computed tomography)-based staging vs. patients with conventional staging. Numbers of patients (percentage) are presented. Pearson’s chi-square test was used for comparisons

Parameter	Patients staged with PET-CT, *n* = 170	Patients with conventional staging, *n* = 103	*p*-value
Pneumonitis ≥ grade 1	45 (26.5)	23 (22.3)	0.44
Pneumonitis ≥ grade 2	19 (11.2)	8 (7.8)	0.36
Pneumonitis = grade 3	4 (2.4)	0 (0.0)	0.12
Pneumonitis ≥ grade 4	0 (0.0)	0 (0.0)	–
Esophagitis ≥ grade 1	107 (62.9)	55 (53.4)	0.12
Esophagitis ≥ grade 2	41 (24.1)	15 (14.6)	0.06
Esophagitis = grade 3	15 (8.8)	8 (7.8)	0.76
Esophagitis ≥ grade 4	0 (0.0)	0 (0.0)	–
Skin erythema ≥ grade 1	68 (40.0)	37 (35.9)	0.50
Skin erythema = grade 2	2 (1.2)	1 (1.0)	0.87
Skin erythema ≥ grade 3	0 (0.0)	0 (0.0)	–
Nausea ≥ grade 1	49 (28.8)	35 (34.0)	0.37
Nausea ≥ grade 2	14 (8.2)	9 (8.7)	0.89
Nausea = grade 3	4 (2.4)	3 (2.9)	0.78
Nausea ≥ grade 4	0 (0.0)	0 (0.0)	–
Lung infection ≥ grade 2	44 (25.9)	14 (13.6)	0.02
Lung infection ≥ grade 3	32 (18.8)	8 (7.8)	0.01
Lung infection ≥ grade 4	5 (2.9)	0 (0)	0.08
Lung infection = grade 5	1 (0.6)	0 (0.0)	0.44
Dyspnea ≥ grade 1	107 (62.9)	73 (70.9)	0.18
Dyspnea ≥ grade 2	51 (30.0)	32 (31.1)	0.85
Dyspnea ≥ grade 3	25 (14.7)	15 (14.6)	0.97
Dyspnea ≥ grade 4	0 (0.0)	0 (0.0)	–
Myocardial infarction = grade 2	3 (1.8)	1 (1.0)	0.60
Myocardial infarction ≥ grade 3	0 (0.0)	0 (0.0)	–
Anemia ≥ grade 1	151 (88.8)	92 (89.3)	0.90
Anemia ≥ grade 2	59 (34.7)	26 (25.2)	0.1
Anemia ≥ grade 3	15 (8.8)	6 (5.8)	0.37
Anemia = grade 4	1 (0.6)	0 (0.0)	0.44
Anemia = grade 5	0 (0.0)	0 (0.0)	–
Leukopenia ≥ grade 1	113 (66.5)	50 (48.5)	< 0.01
Leukopenia ≥ grade 2	74 (43.5)	37 (35.9)	0.22
Leukopenia ≥ grade 3	42 (24.7)	20 (19.4)	0.31
Leukopenia = grade 4	14 (8.2)	3 (2.9)	0.08
Leukopenia = grade 5	0 (0.0)	0 (0.0)	–
Thrombocytopenia ≥ grade 1	85 (50.0)	41 (39.8)	0.10
Thrombocytopenia ≥ grade 2	18 (10.6)	11 (10.7)	0.98
Thrombocytopenia ≥ grade 3	8 (4.7)	4 (3.9)	0.75
Thrombocytopenia = grade 4	2 (1.2)	0 (0.0)	0.27
Thrombocytopenia = grade 5	0 (0.0)	0 (0.0)	–

#### Outcomes

**Fig. 2:**
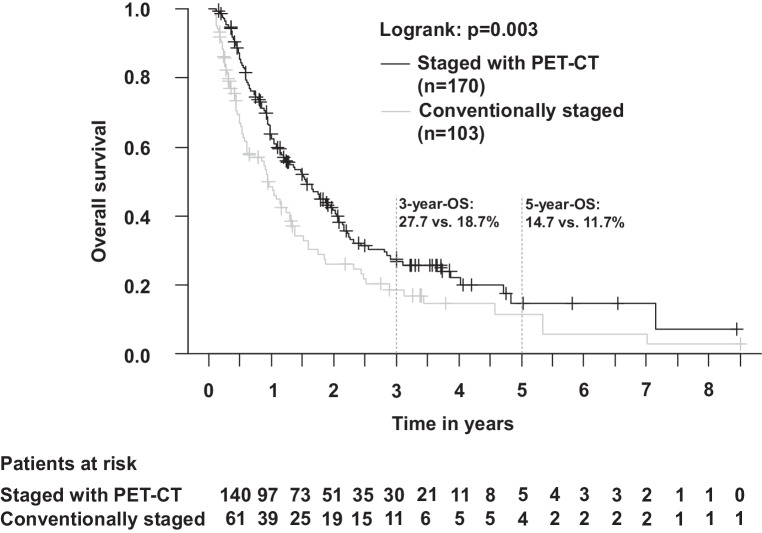
Overall survival in patients with PET-CT(positron emission tomography-computed tomography)-based staging vs. conventional staging (Kaplan–Meier; *p* = 0.003). The 3‑year overall survival (OS) was 27.7% (with PET-CT) vs. 18.7% (without PET-CT)

**Fig. 3:**
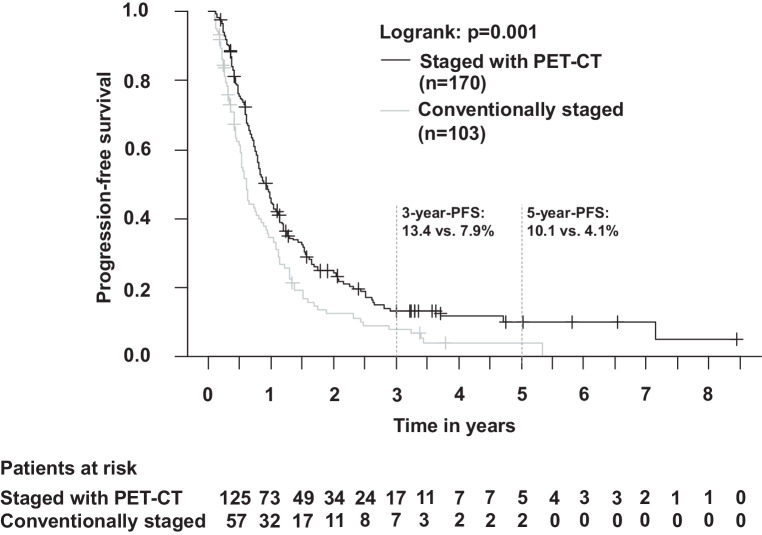
Progression-free survival in patients with PET-CT(positron emission tomography-computed tomography)-based staging vs. conventional staging (Kaplan–Meier; *p* = 0.001). The 3‑year progression-free survival (PFS) was 13.4% (with PET-CT) vs. 7.9% (without PET-CT)

Outcomes were compared between patients staged with PET-CT vs. conventional staging: OS, PFS, LRPFS, and DPFS were superior with PET-CT-based staging (Suppl. Table S3, univariable Cox regression analysis; Figs. 2 and 3, survival curves for OS and PFS). Statistical significance was retained in multivariable analysis (Table 3, inclusion of parameters with significant influence [*p* < 0.05] on OS, PFS, LRPFS, or DPFS in univariable analysis). Parameters with significant influence on survival in multivariable analysis included age (OS), Karnofsky index (OS, PFS, LRPFS, DPFS), and applied RT dose (OS, PFS, LRPFS, DPFS). Please see Figs. 2 and 3 for OS and PFS. The 2‑year LRPFS was 27.2% (PET-CT-based staging) vs. 14.4% (conventional staging; *p* < 0.05). The 2‑year DPFS was 32.3% (PET-CT-based staging) vs. 18.6% (conventional staging; *p* < 0.05). In patients with PET-CT, death was documented for 114/170 (67.1%) patients. In patients with conventional staging, death was documented for 76/103 (73.8%) patients. In patients with PET-CT, tumor progression was documented in 77/170 (45.3%) patients, including locoregional recurrences in 58/170 (34.1%) patients and distant progression in 49/170 (28.8%) patients. In patients with conventional staging, tumor progression was documented in 31/103 (30.1%) patients, including locoregional recurrences in 21/103 (20.4%) patients and distant progression in 19/103 (18.4%) patients.

**Table 3:** Outcomes in patients with PET-CT(positron emission tomography-computed tomography)-based staging vs. conventional staging including further parameters with a possible influence on survival. Multivariable Cox regression analysis. For pre-selection of parameters, please see Suppl. Table S3 (univariable analysis; inclusion of parameters with significant influence [*p* < 0.05] on OS, PFS, LRPFS, or DPFS in the presented multivariable analysis)

Parameter	OS		PFS		LRPFS		DPFS	
	Hazard ratio (95% confidence interval)	*p*-value	Hazard ratio (95% confidence interval)	*p*-value	Hazard ratio (95% confidence interval)	*p*-value	Hazard ratio (95% confidence interval)	*p*-value
PET-CT staging (*n* = 170) vs. conventional staging (*n* = 103)	0.7 (0.52–0.94)	0.02	0.7 (0.54–0.93)	0.01	0.7 (0.53–0.92)	0.01	0.73 (0.56–0.97)	0.03
Gender female (*n* = 66) vs. male (*n* = 207)	0.77 (0.54–1.1)	0.15	0.76 (0.56–1.05)	0.1	0.76 (0.55–1.05)	0.1	0.86 (0.62–1.19)	0.36
Age ≥ 65 years (*n* = 170) vs. < 65 years (*n* = 103)	1.6 (1.17–2.2)	< 0.01	1.15 (0.87–1.52)	0.34	1.29 (0.97–1.72)	0.08	1.23 (0.92–1.64)	0.16
Charlson comorbidity index (median = 4) ≥ median (*n* = 160) vs. < median (*n* = 113)	1.14 (0.78–1.66)	0.5	0.95 (0.68–1.34)	0.78	0.9 (0.63–1.28)	0.55	1.07 (0.75–1.53)	0.71
Karnofsky index (median = 90) ≥ median (*n* = 154) vs. < median (*n* = 119)	0.67 (0.5–0.9)	< 0.01	0.7 (0.54–0.91)	< 0.01	0.69 (0.52–0.9)	< 0.01	0.74 (0.57–0.98)	0.03
Applied dose > 60 Gy (*n* = 125) vs. ≤ 60 Gy (*n* = 148)	0.64 (0.48–0.87)	< 0.01	0.62 (0.48–0.82)	< 0.001	0.65 (0.49–0.86)	< 0.01	0.74 (0.56–0.98)	< 0.001
Radiochemotherapy (*n* = 223) vs. radiotherapy only (*n* = 50)	0.83 (0.57–1.22)	0.35	0.96 (0.67–1.37)	0.81	0.96 (0.67–1.38)	0.82	0.86 (0.6–1.23)	0.4

*OS* overall survival, *PFS* progression-free survival, *LRPFS* locoregional progression-free survival, *DPFS* distant progression-free survival

### IMRT/VMAT vs. 3D-CRT

#### Baseline, clinical, and treatment characteristics

Parameters were compared in patients with IMRT/VMAT (*n* = 99) vs. 3D-CRT (*n* = 64; Table 4). Patients underwent IMRT/VMAT vs. 3D-CRT more frequently in the later (12/2013–12/2019, 76.8% vs. 9.4% of the patients) than in the earlier (01/2008–11/2013, 23.2% vs. 90.6% of the patients) treatment period (*p* < 0.01). In patients with IMRT/VMAT vs. 3D-CRT, we found higher applied RT doses (median 66 Gy vs. 60 Gy; *p* = 0.03), a lower percentage of the lungs receiving ≥ 40 Gy (V40Gy [%]; *p* < 0.01), and a higher percentage of the lungs exposed to lower RT doses (V10Gy [%] and V5Gy [%]; each *p* < 0.01).

**Table 4:** Comparison of baseline, clinical, and treatment characteristics in patients with IMRT/VMAT (intensity-modulated radiotherapy/volumetric modulated arc therapy) vs. 3D-CRT (3D-conformal radiotherapy). Median (minimum–maximum) values or numbers of patients (percentage) are presented, if not otherwise specified

Parameter	IMRT/VMAT, *n* = 99	3D-CRT, *n* = 64	*p*-value
Age	68.4 (39.2–89.2)	68.1 (32.5–86.2)	0.34^e^
Female	25 (25.3)	12 (18.8)	0.33^f^
Male	74 (74.7)	52 (81.2)	–
Karnofsky index	90 (50–90)	90 (50–90)	0.25^e^
Charlson comorbidity index	4 (0–14)	4 (0–10)	0.07^e^
Stage cT1–2	38 (38.4)	16 (25.0)	0.08^f^
Stage cT3–4	61 (61.6)	48 (75.0)	–
Stage cN0–1	11 (11.1)	10 (15.6)	0.4^f^
Stage cN2–3	88 (88.9)	54 (84.4)	–
Squamous cell carcinoma	60 (60.6)	37 (57.8)	0.73^e^
Adenocarcinoma	36 (36.4)	25 (39.1)	–
Other types^a^	3 (3)	2 (3.1)	
Earlier treatment period, 01/2008–11/2013^b^	23 (23.2)	58 (90.6)	< 0.01^f^
Later treatment period, 12/2013–12/2019^c^	76 (76.8)	6 (9.4)	–
Radiotherapy only	20 (20.2)	9 (14.1)	0.32^f^
Radiochemotherapy	79 (79.8)	55 (85.9)	–
Concomitant cisplatin/vinorelbine	40 (50.6)	19 (34.5)	0.09^e^
Concomitant low-dose cisplatin	31 (39.2)	29 (52.7)	–
Other type of chemotherapy^d^	8 (10.2)	7 (12.8)	
Consolidation durvalumab	3 (3.0)	1 (1.6)	0.55^f^
Radiotherapy applied dose [Gy]	66 (14–66.6)	60 (10–66.6)	0.03^e^
Radiotherapy completed	86 (86.9)	53 (82.8)	0.48^f^
Radiotherapy ≥ 80% of the planned dose applied	91 (91.9)	58 (90.6)	0.77^f^
Clinical target volume [ml]	237.9 (32.8–1025.9)	242 (18.4–1236)	0.84^e^
Planning target volume [ml]	732.6 (263.8–2244.1)	729.1 (152.7–2089)	0.44^e^
Lungs mean dose [Gy]	18.2 (4.4–26.1)	17.6 (2.9–27.9)	0.62^e^
Lungs V40Gy [%]	15.7 (0–32.1)	19.4 (0–37.3)	< 0.01^e^
Lungs V30Gy [%]	22.9 (0–38.6)	23.8 (0–42.8)	0.16^e^
Lungs V20Gy [%]	31.8 (0–48.8)	30.4 (0–48.5)	0.52^e^
Lungs V10Gy [%]	51.7 (14.6–85.9)	46.2 (8.2–70.3)	< 0.01^e^
Lungs V5Gy [%]	67.6 (30–99.5)	57.5 (19.5–91)	< 0.001^e^

^a^Further histologic types: not otherwise specified, *n* = 3; pleomorphic type, *n* = 1; adenoid papillary differentiation, *n* = 1

^b^Initiation of radiotherapy/radiochemotherapy from 01/2008–11/2013 (allocation: median of the whole study group)

^c^Initiation of radiotherapy/radiochemotherapy from 12/2013–12/2019 (allocation: median of the whole study group)

^d^Other chemotherapy: concomitant chemotherapy, *n* = 4; cisplatin/pemetrexed, *n* = 1; carboplatin/paclitaxel, *n* = 1; cisplatin/etoposide, *n* = 1; vinorelbine/etoposide, *n* = 1; sequential chemotherapy, *n* = 11; cisplatin/docetaxel, *n* = 4; carboplatin/paclitaxel, *n* = 2; cisplatin/pemetrexed, *n* = 2; cisplatin/vinorelbine, *n* = 1; docetaxel/nintedanib, *n* = 1; pemetrexed, *n* = 1

^e^Kruskal–Wallis test

^f^Pearson’s chi-square test

#### Toxicities

Toxicities were compared between patients with IMRT/VMAT vs. 3D-CRT (Table 5). Rates of pneumonitis ≥ grade 2 (*p* = 0.006), skin erythema ≥ grade 1 (*p* = 0.01), and nausea ≥ grade 2 (*p* = 0.045) were lower with IMRT/VMAT.

**Table 5:** Comparison of toxicities in patients with IMRT/VMAT (intensity-modulated radiotherapy/volumetric modulated arc therapy) vs. 3D-CRT (3D-conformal radiotherapy). Numbers of patients (percentage) are presented. Pearson’s chi-square test was used for comparisons

Parameter	IMRT/VMAT, *n* = 99	3D-CRT, *n* = 64	*p*-value
Pneumonitis ≥ grade 1	22 (22.2)	21 (32.8)	0.13
Pneumonitis ≥ grade 2	6 (6.1)	13 (20.3)	0.006
Pneumonitis = grade 3	3 (3.0)	2 (3.1)	0.97
Pneumonitis ≥ grade 4	0 (0.0)	0 (0.0)	–
Esophagitis ≥ grade 1	59 (59.6)	46 (71.9)	0.11
Esophagitis ≥ grade 2	26 (26.3)	17 (26.6)	0.97
Esophagitis = grade 3	8 (8.1)	7 (10.9)	0.54
Esophagitis ≥ grade 4	0 (0.0)	0 (0.0)	–
Skin erythema ≥ grade 1	31 (31.3)	33 (51.6)	0.01
Skin erythema = grade 2	1 (1.0)	1 (1.6)	0.75
Skin erythema ≥ grade 3	0 (0.0)	0 (0.0)	–
Nausea ≥ grade 1	32 (32.3)	19 (29.7)	0.72
Nausea ≥ grade 2	6 (6.1)	10 (15.6)	0.045
Nausea = grade 3	3 (3.0)	2 (3.1)	0.66
Nausea ≥ grade 4	0 (0.0)	0 (0.0)	–
Lung infection ≥ grade 2	25 (25.3)	17 (26.6)	0.85
Lung infection ≥ grade 3	18 (18.2)	13 (20.3)	0.76
Lung infection ≥ grade 4	2 (2.0)	3 (4.7)	0.33
Lung infection = grade 5	1 (1.0)	0 (0.0)	0.42
Dyspnea ≥ grade 1	63 (63.6)	42 (65.6)	0.80
Dyspnea ≥ grade 2	34 (34.3)	19 (29.7)	0.54
Dyspnea = grade 3	18 (18.2)	5 (7.8)	0.06
Dyspnea ≥ grade 4	0 (0.0)	0 (0.0)	–
Myocardial infarction ≥ grade 2	2 (2.0)	1 (1.6)	0.83
Myocardial infarction = grade 3	1 (1.0)	0 (0.0)	0.41
Myocardial infarction ≥ grade 4	0 (0.0)	0 (0.0)	–
Anemia ≥ grade 1	86 (86.9)	57 (89.1)	0.68
Anemia ≥ grade 2	38 (38.4)	18 (28.1)	0.18
Anemia ≥ grade 3	12 (12.1)	4 (6.3)	0.22
Anemia = grade 4	1 (1.0)	0 (0.0)	0.42
Anemia = grade 5	0 (0.0)	0 (0.0)	–
Leukopenia ≥ grade 1	66 (66.7)	38 (59.4)	0.34
Leukopenia ≥ grade 2	44 (44.4)	24 (37.5)	0.38
Leukopenia ≥ grade 3	24 (24.2)	15 (23.4)	0.91
Leukopenia = grade 4	9 (9.1)	3 (4.7)	0.29
Leukopenia = grade 5	0 (0.0)	0 (0.0)	–
Thrombocytopenia ≥ grade 1	53 (53.1)	29 (45.3)	0.31
Thrombocytopenia ≥ grade 2	11 (11.1)	7 (10.9)	0.97
Thrombocytopenia ≥ grade 3	5 (5.1)	3 (4.7)	0.93
Thrombocytopenia = grade 4	1 (1.0)	2 (3.1)	0.33
Thrombocytopenia = grade 5	0 (0.0)	0 (0.0)	–

#### Outcomes

**Fig. 4:**
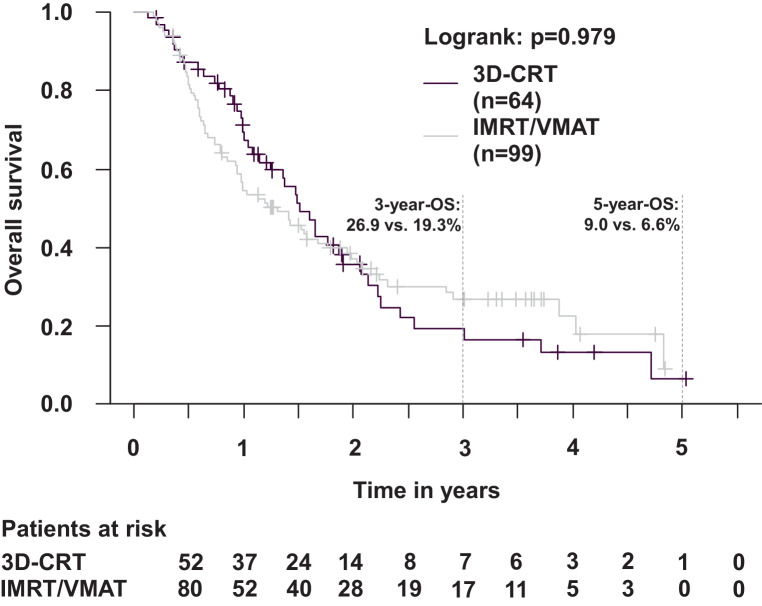
Overall survival in patients with IMRT/VMAT (intensity-modulated radiotherapy/volumetric modulated arc therapy) vs. 3D-CRT (3D-conformal radiotherapy) (Kaplan–Meier; *p* = 0.979). The 3‑year overall survival (OS) was 26.9% (IMRT/VMAT) vs. 19.3% (3D-CRT)

**Fig. 5:**
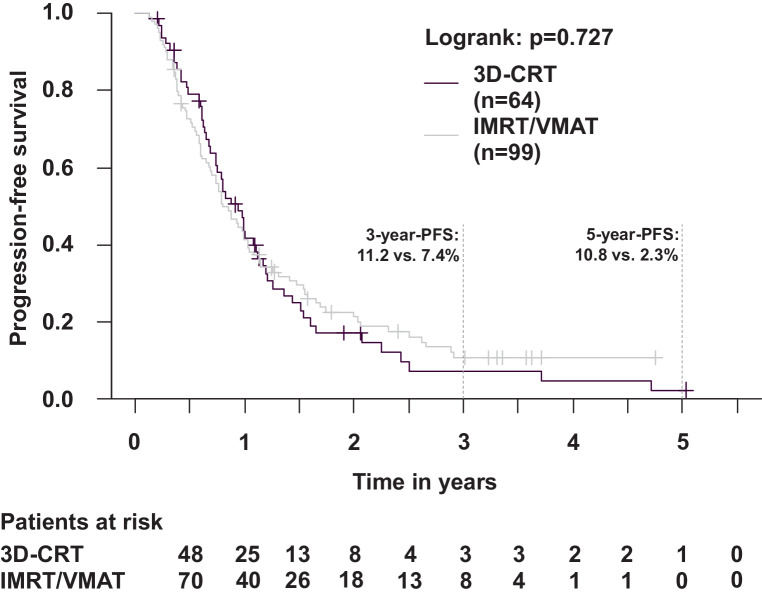
Progression-free survival in patients with IMRT/VMAT (intensity-modulated radiotherapy/volumetric modulated arc therapy) vs. 3D-CRT (3D-conformal radiotherapy) (Kaplan–Meier; *p* = 0.727). The 3‑year progression-free survival (PFS) was 11.2% (IMRT/VMAT) vs. 7.4% (3D-CRT)

Outcomes were compared between patients with IMRT/VMAT vs. 3D-CRT. There were no differences in OS (*p* = 0.98; Fig. 4), PFS (*p* = 0.73; Fig. 5), LRPFS (*p* = 0.94), and DPFS (*p* = 0.44). The 3‑year OS was 26.9% (IMRT/VMAT) vs. 19.3% (3D-CRT), the 3‑year PFS was 11.2% (IMRT/VMAT) vs. 7.4% (3D-CRT), the 2‑year LRPFS was 24.6% (IMRT/VMAT) vs. 20.1% (3D-CRT), and the 2‑year DPFS was 30.9% (IMRT/VMAT) vs. 21.3% (3D-CRT). In patients with IMRT/VMAT, death was documented for 69/99 (69.7%) patients. In patients with 3D-CRT, death was documented for 43/64 (67.2%) patients. In patients with IMRT/VMAT, tumor progression was documented in 43/99 (43.4%) patients, including locoregional recurrences in 35/99 (35.4%) patients and distant progression in 26/99 (26.3%) patients. In patients with 3D-CRT, tumor progression was documented in 31/64 (48.4%) patients, including locoregional recurrences in 20/64 (31.3%) patients and distant progression in 24/64 (37.5%) patients.

## Discussion

During the past two decades, relevant progress has been achieved in multimodal treatment strategies for the management of localized NSCLC [25]. The recent introduction of consolidation durvalumab yielded 5‑year survival rates of 42.9% with RCT–immunotherapy in stage III [26, 27]. Boys et al. studied 126 patients with definitive RCT for stage III NSCLC and evaluated the eligibility for durvalumab consolidation in clinical practice [28]. A total of 56/126 patients (44.4%) were ineligible for consolidation [28]. The reasons included progressive disease or death (*n* = 14; 25%), RT dose < 54 Gy (*n* = 9; 16%), and radiation pneumonitis ≥ grade 2 (*n* = 8; 14%) [28]. Thus, optimization of RCT with precise target volume definition, sufficient RT dose, and avoidance of toxicities potentially has a beneficial influence and, thus, is of utmost relevance for the efficacy of multimodal treatment [29, 30]. Before the advent of immunotherapy in the treatment of stage III NSCLC, in a cohort study from 2010–2018 in Denmark, Meldgaard et al. reported improved outcomes in the more contemporary study period of 2016–2018 [2]. These improvements certainly reflect, to a major extent, advances in imaging modalities, RT planning, and RT techniques [5, 25]. In 2020, The German PET-Plan trial prospectively compared PET-CT-based planning vs. conventional planning in definitive RCT for NSCLC [6, 31]. Nestle et al. reported a potential for improved local control and good feasibility [6]. In 2017, in a secondary analysis of the RTOG 0617 study, Chun et al. found lower rates of toxicities with a dynamic RT technique (IMRT) [5]. Herein, we studied the specific clinical outcomes of patients who were treated with vs. without these refined strategies (PET-CT-based staging and dynamic RT techniques [IMRT/VMAT]) from 2008 to 2019.

In this study, PET-CT-staged patients presented with lower T stages (cT1–2 vs. cT3–4; *p* = 0.049) than conventionally staged patients. Comparably, when Vokes et al. analyzed the impact of PET-CT on survival in 598 NSCLC patients, stage IIIA (vs. IIIB) was diagnosed in 49% of the patients with PET-CT vs. 39% of patients without PET-CT [32]. The differences in the presented groups can be explained by our study design. We included patients who were referred to the radiation oncology department for curative RT/RCT. However, PET-CT vs. conventional CT-based staging can detect additional metastases in up to 10% of patients [33]. Thus, in patients undergoing PET-CT, upstaging might have led to a stage shift from localized cT3–4 tumors (Hao et al.; association of T stages with rates of metastasis [34]) to UICC stage IV. This might explain the overrepresentation of T1–2 patients relative to T3–4 patients in the current PET-CT study group [35].

When compared to conventionally staged patients, PET-CT-staged patients received higher RT doses (median 66 Gy vs. 60 Gy; maximum dose 70 Gy; *p* < 0.01), and had higher RT completion rates (87.1% vs. 77.7%; *p* = 0.04; ≥ 80% of the planned dose applied: 93.5% vs. 82.5%; *p* < 0.01). Additionally, with only a trend towards statistical significance, PET-CT-staged patients had higher rates of RCT vs. RT only (PET-CT: 84.7%; conventional: 76.7%; *p* = 0.1) and, in patients who received RCT, with concomitant cisplatin/vinorelbine vs. low-dose cisplatin (PET-CT: 41.7%; conventional: 25.3%; *p* = 0.08). Current studies aim at treatment intensification via RT dose escalation in adequately selected patients, tumor subsets, and treatment volumes and via optimization of systemic treatment [36]. Here, we have demonstrated relevant improvements in the PET-CT-staged study group. At the same time, rates of lung infections (≥ grade 2, *p* = 0.02; ≥ grade 3, *p* = 0.01) and leukopenia (≥ grade 1, *p* < 0.01) were higher in PET-CT-staged patients. These findings might be explained by intensified treatment in this group (previous studies; increased rates of hematologic toxicities/leukopenia with RCT vs. RT [37] and with cisplatin/vinorelbine vs. low-dose cisplatin [7, 38]; neutropenia as an important risk factor for infections [24]). In the present study, these relations were partly demonstrated for patients with leukopenia (see “Toxicities”; higher proportion of patients with RCT vs. RT only, with ≥ 80% of the planned RT dose applied, and with a higher applied RT dose). However, putatively due to the retrospective study design including patients within a long period of time, there were no significant differences in parameters for patients with lung infections. The coincidence of improved outcomes and higher toxicity rates in PET-CT-staged patients emphasizes the need for a detailed risk–benefit assessment during planning and application of treatment modalities [39].

Patients with PET-CT vs. conventional staging experienced better OS, PFS, LRPFS, and DPFS. Comparably, Vokes et al. found improved survival with PET-CT vs. conventional staging in RCT for localized NSCLC [32]. The influence on survival might, to a certain extent, be attributed to the detection of occult metastases and stage migration (in the presented study, possibly leading to overrepresentation of T1–2 patients in the PET-CT group, please see above) [32, 40]. The multivariable model with inclusion of parameters with a possible influence on outcomes was established to take these aspects into account. Remarkably, the survival advantages in PET-CT-staged patients were retained in multivariable analysis. Additionally, we found an association of previously reported baseline parameters (age [7, 41], Karnofsky index/performance status [42]) and of intensified treatment (applied RT dose > 60 Gy vs. ≤ 60 Gy; maximum dose of 70 Gy [43]) with survival. Taken together, with positive selection of PET-CT patients via detection of occult metastases, it can be concluded that PET-CT is an important basis for optimal RT/RCT indication setting [32, 40].

PET-CT-staged patients were treated more frequently in the more contemporary time period (12/2013–12/2019 vs. 01/2008–11/2013) when compared to conventionally staged patients. These findings reflect the increasing application of PET-CT in clinical routine (Bedir et al.: 2007–2010, initial implementation of PET-CT; 2011–2014, era in between; 2015–2018, PET-CT widely available and used) [44]. Next, patients in the PET-CT group were irradiated more frequently with modern RT techniques (IMRT/VMAT vs. 3D-CRT) when compared to conventionally staged patients. This reflects distinct parallels of RT technique and PET-CT introduction and further implementation in the clinical routine of NSCLC treatment [44].

In the presented study, we found a higher RT dose with IMRT/VMAT vs. 3D-CRT (median 66 Gy vs. 60 Gy; *p* = 0.03). Additionally, IMRT/VMAT-treated patients had higher rates (trend, not statistically significant) of intensified chemotherapy (concomitant cisplatin/vinorelbine: IMRT/VMAT, 50.6%, 3D-CRT, 34.5%, vs. low-dose cisplatin: IMRT/VMAT, 39.2%, 3D-CRT, 52.7%, see Table 4 for other types of chemotherapy, p=0.09). Comparable with the influence of PET-CT-based staging, this provides evidence that modern RT techniques enable treatment intensification. In contrast to findings with PET-CT, there were no effects of IMRT/VMAT on survival (OS, PFS, LRPFS, DPFS). Outcomes were comparable with IMRT/VMAT (3-year OS 26.9%) vs. 3D-CRT (3-year OS 19.3%). These results are in line with previous studies (no differences in OS, PFS, local failure, distant metastases) [5]. Chun et al. discussed that—via advantages in heart or lung sparing—dynamic RT techniques could be associated with superior survival in long-term outcomes [5]. However, a recent press release indicated that there were no differences at 5‑year follow-up [45].

Furthermore, with IMRT/VMAT vs. 3D-CRT, we found reduced lung exposure at higher dose levels (V40Gy) and increased exposure at lower dose levels (V5Gy and V10Gy; each, *p* < 0.01). These findings are as expected (general increase in low-dose bath with dynamic RT techniques [46]) and comparable with a planning study by Li et al. (higher dose levels, V30Gy, advantages of IMRT/VMAT; lower dose levels, V5Gy, highest exposure with VMAT) [47]. In line with previous studies [5], we found lower rates of pneumonitis with IMRT/VMAT (≥ grade 2, *p* < 0.01). Additionally, radiation dermatitis was reduced with IMRT/VMAT (≥ grade 1, *p* = 0.01). Reduced RT-induced skin reactions with dynamic RT techniques have previously been described for different tumor entities (IMRT vs. 3D-CRT in breast cancer [48], VMAT vs. 3D-CRT in anal cancer [49], and VMAT vs. 3D-CRT in rectal cancer [50]). Interestingly, in spite of more intensified systemic treatment in IMRT/VMAT-treated vs. 3D-CRT-treated patients (previous studies: higher rates of nausea with cisplatin/vinorelbine [7]; presented study: trend towards higher rates of concomitant cisplatin/vinorelbine vs. low-dose cisplatin), rates of nausea were lower with IMRT/VMAT (≥ grade 2, *p* = 0.045). Since IMRT/VMAT was more frequently applied in the later period (12/2013–12/2019 vs. 01/2008–11/2013), it might be hypothesized that general optimization of antiemetic treatment could have contributed to lower rates of nausea (first American Society of Clinical Oncology [ASCO] guideline on antiemetics published in 1999; updates in 2006, 2011, 2015, 2017, and 2020 [51]).

Finally, the limitations of the presented study are discussed. RT with IMRT/VMAT and staging with PET-CT can be considered as a current standard of care for NSCLC patients. Thus, conventional staging and 3D-CRT should not be regularly applied. At the same time, in clinical routine (e.g., when PET-CT is not available timely in patients with a high tumor burden who require urgent RT), PET-CT will not be realizable in certain patients. Additionally, we present a study with a detailed analysis of patient characteristics and clinical outcomes. Thus, the presented study adds relevant information on personalized treatment in localized NSCLC.

## Conclusion

Refined RT/RCT strategies with PET-CT and IMRT/VMAT enable intensification of multimodal treatment. Reduction of toxicities with a refined RT technique widens the therapeutic window. The coincidence of improved outcomes and higher toxicity rates in PET-CT-staged patients emphasizes the need for a detailed risk–benefit assessment during planning and application of treatment modalities. The presented real-world data serve as an important basis in the changing landscape with current RCT–immunotherapy strategies for localized NSCLC. Specifically, patient eligibility for durvalumab consolidation after RCT could be increased with refined strategies.

## Supplementary Information

**Suppl. Table S1. **Comparison of baseline, clinical, and treatment characteristics in patients with lung infection ≥ grade 3 vs. patients without lung infection ≥ grade 3. Median (minimum–maximum) values or numbers of patients (percentage) are presented, if not otherwise specified. ^1^ Initiation of radiotherapy/radiochemotherapy from 01/2008–11/2013 (allocation: median of the whole study group). ^2^ Initiation of radiotherapy/radiochemotherapy from 12/2013–12/2019 (allocation: median of the whole study group). ^3^ Pearson’s chi-square test. ^4^ Kruskal–Wallis test.

**Suppl. Table S2. **Comparison of baseline, clinical, and treatment characteristics in patients with leukopenia ≥ grade 1 vs. patients without leukopenia ≥ grade 1. Median (minimum–maximum) values or numbers of patients (percentage) are presented, if not otherwise specified. ^1^ Initiation of radiotherapy/radiochemotherapy from 01/2008–11/2013 (allocation: median of the whole study group). ^2^ Initiation of radiotherapy/radiochemotherapy from 12/2013–12/2019 (allocation: median of the whole study group). ^3^ Pearson’s chi-square test. ^4^ Kruskal–Wallis test.

**Suppl. Table S3.** Outcomes in patients with PET-CT-based staging vs. conventional staging including further parameters with possible influence on survival. Univariable Cox regression analysis. *OS* overall survival, *PFS* progression-free survival, *LRPFS* locoregional progression-free survival, *DPFS* distant progression-free survival.

## Data Availability

The datasets generated and/or analyzed during the current study are available from the corresponding author upon reasonable request.
