# Supplementary material for: Treatment of non-small cell lung cancer: advances following the introduction of PET-CT and IMRT/VMAT
Source: Strahlenther Onkol. 2025 Mar 6;201(11):1123–36. doi: 10.1007/s00066-025-02377-0 (PMC12546295; doi:10.1007/s00066-025-02377-0)
Supplement: Supplementary file 1 — Suppl. Table S1. Comparison of baseline, clinical, and treatment characteristics in patients with lung infection ≥ grade 3 vs. patients without lung infection ≥ grade 3. Median (minimum–maximum) values or numbers of patients (percentage) are presented, if not otherwise specified. 1 Initiation of radiotherapy/radiochemotherapy from 01/2008–11/2013 (allocation: median of the whole study group). 2 Initiation of radiotherapy/radiochemotherapy from 12/2013–12/2019 (allocation: median of the whole study group). 3 Pearson’s chi-square test. 4 Kruskal–Wallis test. [file 66_2025_2377_MOESM1_ESM.docx]

**Suppl. Table S1.** Comparison of baseline, clinical, and treatment characteristics in patients with lung infection ≥grade 3 vs. patients without lung infection ≥grade 3. Median (minimum-maximum) values or numbers of patients (percentage) are presented, if not otherwise specified. ^1^ Initiation of radiotherapy/radiochemotherapy from 01/2008-11/2013 (allocation, median of the whole study group). ^2^ Initiation of radiotherapy/radiochemotherapy from 12/2013-12/2019 (allocation, median of the whole study group). ^3^ Pearson’s chi-square test. ^4^ Kruskal-Wallis test.

| **Parameter** | **Patients with lung infection ≥grade 3, n=40** | **Patients without lung infection ≥grade 3, n=233** | **p-value** |
| --- | --- | --- | --- |
| Stage cT1-2 | 11 (27.5) | 69 (29.6) | 0.79 ^3^ |
| Stage cT3-4 | 29 (72.5) | 164 (70.4) |  |
| Earlier treatment period, 01/2008-11/2013 ^1^ | 20 (50.0) | 116 (49.8) | 0.98 ^3^ |
| Later treatment period, 12/2013-12/2019 ^2^ | 20 (50.0) | 117 (50.2) |  |
| Radiotherapy technique, ≥80% of the course with intensity modulated radiotherapy (IMRT)/volumetric modulated arc therapy (VMAT) | 18 (45.0) | 93 (39.9) | 0.49 ^4^ |
| Radiotherapy technique, ≥80% of the course with 3-dimensional conformal radiotherapy (3D-CRT) | 21 (52.5) | 130 (55.8) |  |
| Radiotherapy technique, no major technique (neither IMRT/VMAT nor 3D-CRT, cut-off, ≥80%).) | 1 (2.5) | 10 (4.3) |  |
| Radiotherapy only | 7 (17.5) | 43 (18.5) | 0.89 ^3^ |
| Radiochemotherapy | 33 (82.5) | 190 (81.5) |  |
| Concomitant  cisplatin/vinorelbine | 11 (33.3) | 69 (36.3) | 0.53 ^4^ |
| Concomitant  low-dose cisplatin | 19 (57.6) | 106 (55.8) |  |
| Other type of  chemotherapy | 3 (9.1) | 15 (7.9) |  |
| Radiotherapy, applied dose [Gy] | 60.0 (10.0-70.0) | 60.0 (2.0-70.0) | 0.33 ^4^ |
| Radiotherapy completed | 31 (77.5) | 197 (84.5) | 0.27 ^3^ |
| Radiotherapy, ≥80% of the planned dose applied | 35 (87.5) | 209 (89.7) | 0.68 ^3^ |
| Leukopenia, ≥grade 1 | 22 (55.0) | 141 (60.5) | 0.51 ^3^ |
| Leukopenia, ≥grade 2 | 16 (40.0) | 95 (40.8) | 0.93 ^3^ |
| Leukopenia, ≥grade 3 | 8 (20.0) | 54 (23.2) | 0.66 ^3^ |
| Leukopenia, ≥grade 4 | 2 (5.0) | 15 (6.4) | 0.73 ^3^ |
