# Supplementary material for: Treatment of non-small cell lung cancer: advances following the introduction of PET-CT and IMRT/VMAT
Source: Strahlenther Onkol. 2025 Mar 6;201(11):1123–36. doi: 10.1007/s00066-025-02377-0 (PMC12546295; doi:10.1007/s00066-025-02377-0)
Supplement: Supplementary file 2 — Suppl. Table S2. Comparison of baseline, clinical, and treatment characteristics in patients with leukopenia ≥ grade 1 vs. patients without leukopenia ≥ grade 1. Median (minimum–maximum) values or numbers of patients (percentage) are presented, if not otherwise specified. 1 Initiation of radiotherapy/radiochemotherapy from 01/2008–11/2013 (allocation: median of the whole study group). 2 Initiation of radiotherapy/radiochemotherapy from 12/2013–12/2019 (allocation: median of the whole study group). 3 Pearson’s chi-square test. 4 Kruskal–Wallis test. [file 66_2025_2377_MOESM2_ESM.docx]

**Suppl. Table S2.** Comparison of baseline, clinical, and treatment characteristics in patients with leukopenia ≥grade 1 vs. patients without leukopenia ≥grade 1. Median (minimum-maximum) values or numbers of patients (percentage) are presented, if not otherwise specified. ^1^ Initiation of radiotherapy/radiochemotherapy from 01/2008-11/2013 (allocation, median of the whole study group). ^2^ Initiation of radiotherapy/radiochemotherapy from 12/2013-12/2019 (allocation, median of the whole study group). ^3^ Pearson’s chi-square test. ^4^ Kruskal-Wallis test.

| **Parameter** | **Patients with leukopenia ≥grade 1, n=163** | **Patients without leukopenia ≥grade 1, n=110** | **p-value** |
| --- | --- | --- | --- |
| Stage cT1-2 | 47 (28.8) | 33 (30.0) | 0.84 ^3^ |
| Stage cT3-4 | 116 (71.2) | 77 (70.0) |  |
| Earlier treatment period, 01/2008-11/2013 ^1^ | 67 (41.1) | 69 (62.7) | <0.01 ^3^ |
| Later treatment period, 12/2013-12/2019 ^2^ | 96 (58.9) | 41 (37.3) |  |
| Radiotherapy technique, ≥80% of the course with intensity modulated radiotherapy (IMRT)/volumetric modulated arc therapy (VMAT) | 74 (45.4) | 37 (33.6) | 0.15 ^4^ |
| Radiotherapy technique, ≥80% of the course with 3-dimensional conformal radiotherapy (3D-CRT) | 80 (49.1) | 71 (64.5) |  |
| Radiotherapy technique, no major technique (neither IMRT/VMAT nor 3D-CRT, cut-off, ≥80%).) | 9 (5.5) | 2 (1.8) |  |
| Radiotherapy only | 12 (7.4) | 38 (34.5) | <0.01 ^3^ |
| Radiochemotherapy | 151 (92.6) | 72 (65.5) |  |
| Concomitant  cisplatin/vinorelbine | 60 (39.7) | 20 (27.8) | 0.09 ^4^ |
| Concomitant  low-dose cisplatin | 80 (53.0) | 45 (62.5) |  |
| Other type of  chemotherapy | 11 (7.3) | 7 (9.7) |  |
| Radiotherapy, applied dose [Gy] | 62.0 (16.0-70.0) | 60.0 (2.0-70.0) | <0.01 ^4^ |
| Radiotherapy completed | 142 (87.1) | 86 (78.2) | 0.05 ^3^ |
| Radiotherapy, ≥80% of the planned dose applied | 153 (93.9) | 91 (82.7) | <0.01 ^3^ |
