# Supplementary material for: Treatment of non-small cell lung cancer: advances following the introduction of PET-CT and IMRT/VMAT
Source: Strahlenther Onkol. 2025 Mar 6;201(11):1123–36. doi: 10.1007/s00066-025-02377-0 (PMC12546295; doi:10.1007/s00066-025-02377-0)
Supplement: Supplementary file 3 — Suppl. Table S3. Outcomes in patients with PET-CT-based staging vs. conventional staging including further parameters with possible influence on survival. Univariable Cox regression analysis. OS overall survival, PFS progression-free survival, LRPFS locoregional progression-free survival, DPFS distant progression-free survival. [file 66_2025_2377_MOESM3_ESM.docx]

**Suppl. Table S3.** Outcomes in patients with PET-CT-based staging vs. conventional staging including further parameters with possible influence on survival. Univariable Cox regression analysis. OS—overall survival. PFS—progression-free survival. LRPFS—locoregional progression-free survival. DPFS—distant progression-free survival.

| **Parameter** | **OS** | | **PFS** | | **LRPFS** | | **DPFS** | |
| --- | --- | --- | --- | --- | --- | --- | --- | --- |
|  | **Hazard ratio (95% confidence interval)** | **p-value** | **Hazard ratio (95% confidence interval)** | **p-value** | **Hazard ratio (95% confidence interval)** | **p-value** | **Hazard ratio (95% confidence interval)** | **p-value** |
| PET-CT-based staging (170) vs. conventional staging (103) | 0.65 (0.49-0.87) | <0.01 | 0.65 (0.5-0.85) | <0.01 | 0.65 (0.5-0.86) | <0.01 | 0.66 (0.5-0.86) | <0.01 |
| Gender, female (n=66) vs. male (n=207) | 0.71 (0.5-1.01) | 0.053 | 0.74 (0.54-1.01) | 0.06 | 0.71 (0.51-0.98) | 0.04 | 0.8 (0.58-1.1) | 0.17 |
| Age, ≥65 years (n=170) vs. <65 years (n=103) | 1.61 (1.19-2.19) | <0.01 | 1.15 (0.88-1.5) | 0.31 | 1.33 (1.01-1.76) | 0.04 | 1.17 (0.89-1.55) | 0.27 |
| Charlson Comorbidity Index (median=4), ≥median (n=160) vs. <median (n=113) | 1.54 (1.14-2.07) | <0.01 | 1.11 (0.85-1.45) | 0.44 | 1.18 (0.9-1.54) | 0.24 | 1.22 (0.93-1.61) | 0.15 |
| Karnofsky Index (median=90), ≥median (n=154) vs. <median (n=119) | 0.61 (0.46-0.82) | <0.01 | 0.69 (0.53-0.89) | <0.01 | 0.64 (0.49-0.83) | <0.01 | 0.74 (0.56-0.97) | 0.03 |
| cT3-4 (n=193) vs. cT1-2 (n=80) | 1.2 (0.88-1.65) | 0.26 | 1.18 (0.89-1.57) | 0.25 | 1.13 (0.85-1.51) | 0.4 | 1.18 (0.88-1.59) | 0.27 |
| cN2-3 (n=214) vs. cN0-1 (n=59) | 1.09 (0.94-1.27) | 0.27 | 1.06 (0.92-1.21) | 0.43 | 1.05 (0.91-1.2) | 0.53 | 1.09 (0.94-1.26) | 0.24 |
| Adenocarcinoma (n=96) vs. other histology (n=177) | 0.78 (0.58-1.06) | 0.11 | 0.83 (0.63-1.09) | 0.18 | 0.78 (0.59-1.03) | 0.08 | 0.85 (0.64-1.13) | 0.27 |
| Applied dose, >60Gy (n=125) vs. ≤60Gy (n=148) | 0.62 (0.47-0.83) | <0.01 | 0.57 (0.44-0.74) | <0.001 | 0.61 (0.46-0.8) | <0.001 | 0.56 (0.43-0.74) | <0.001 |
| Radiochemotherapy (n=223) vs. radiotherapy only (n=50) | 0.62 (0.44-0.87) | <0.01 | 0.8 (0.58-1.1) | 0.17 | 0.75 (0.54-1.04) | 0.09 | 0.73 (0.52-1.02) | 0.06 |
| Initiation of treatment from 12/2013-12/2019 (137) vs.  01/2008-11/2013 (136) | 1.02 (0.77-1.36) | 0.88 | 1 (0.77-1.3) | 0.1 | 1.05 (0.8-1.36) | 0.73 | 0.99 (0.75-1.29) | 0.91 |
